# Effects of Rhythmic Gymnastics on Joint Attention and Emotional Problems of Autistic Children: A Preliminary Investigation

**DOI:** 10.1155/2022/2596095

**Published:** 2022-08-10

**Authors:** Guanting Duan, Qing Han, Mingyan Yao, Ran Li

**Affiliations:** College of Physical Education and Sports, Beijing Normal University, Beijing, China

## Abstract

The adaptive rhythmic gymnastics (ARG) course has been specially designed for children with autism spectrum disorders (ASD). The purpose of this study is to discover the influence of the course on the joint attention and emotional problems of ASD children. This study adopted A-B-A cross-subject multibaseline design in a single case research design. The joint attention behaviour of two 6-year-old ASD children was examined. The experiment process was recorded and coded, and the results were analysed. The results illustrated the following: (1) ARG is effective in promoting the development of joint attention in ASD children, but it has a better effect on increasing responding joint attention, and (2) to a certain extent, ARG can boost the classroom participation of ASD children and improve their emotional problems.

## 1. Introduction

It is a remarkably widespread problem that children with autism spectrum disorders (ASD) suffer from a deficit of joint attention. This commonly appears in their early childhood. Joint attention is defined as the behaviour of two people paying attention to an object or event together and sharing an interest in it. It includes two forms, responding joint attention (RJA) and initiating joint attention (IJA). RJA refers to responding to another person's request for joint attention, and IJA signifies the initiative of joint attention and is more inclined to the active behaviours of children [1]. Previous studies have disclosed that the joint attention disorder of ASD children in their early childhood has an unignorable effect on the subsequent development of their language ability [2–4], motor skills (meaning a disciplined way of operating through learning), imitating ability [5], and social communication skills [2, 6], which are viewed as milestones in the development of children's ability to perceive themselves and others [7] and basic abilities of higher-level cognitive and social skills in the theory of psychology.

In recent years, the joint attention of ASD children has increasingly become a research hotspot in the early childhood intervention field. Due to the particularity of the research participants, the single case research method was often used in research design, which involves cross-subject multibaseline design [8–12], multibaseline cross-behaviour experimental design [13], and so on. In terms of research methods, child-centred symbolic play, imitative play, music therapy, and intermediary media, such as video and robots, were generally used to intervene. For example, Kim et al. [14] adopted improvisational music therapy and introduced music as an intervention medium in their research, which allowed children to learn joint attention skills through musical activities. So et al. [15] applied robot-based drama intervention to promote the joint attention and play behaviour of ASD children. Play was used in Wong and Kasari's research [16] as a teaching method to observe the joint attention features of ASD children. According to numerous studies, play is a vital skill for children in their early childhood. Therefore, it was frequently used in intervention studies on the joint attention of ASD children as an intervention method, such as functional play and symbolic play. The play behaviours of ASD children are limited by their stereotyped behaviours [17]. They prefer to engage in simple and repetitive actions. ASD children are less focused than normal children when participating in symbolic games and functional games [18]. Even though, a number of studies in the joint attention intervention of ASD children applied symbolic play. This implies that using the intervention in which children are highly interested can bring the possibility of raising joint attention behaviours. For instance, in a study conducted by D'Ateno et al. [19], a preschooler was shown a video of symbolic play demonstrating actions and was requested to imitate the behaviour in the video. The results indicated that the intervention enhanced the child's speech, attention, and movement reaction. The above shows that play is a good intervention for children.

In previous research, there are limited intervention studies on joint attention of ASD children by exercise. Most of these studies focus on the children's motor skills, social skills, cognitive function, attention, and so on. Compared with children without autism, ASD children are delayed in movement. Conducted an extensive sampling of intellectual function on 101 ASD children, according to the Children's Movement Assessment Group22 (Henderson and Sugden, 1992), 79% of ASD children in this sample have a clear movement disorder, and another 10% are rated as a borderline disorder. Exercise is beneficial to the long-term comprehensive development of children. A large number of studies have disclosed that regular exercise is of great help to the improvement of social interaction disorders and dyskinesias in ASD children. Meanwhile, it also can bring a lot of benefits for the improvement of their cognitive ability [20]. For example, Sowa and Meulenbroek's research [21] conducted a quantitative assessment of ASD children. The research found that regular exercise or exercise intervention has had significant effects on the improvement of social interaction and dyskinesias of ASD children. Moreover, it was discovered that group exercise intervention can help ASD children have greater improvement in their social interaction disorder than individual exercise intervention [21]. Pan [22] pointed out that swimming can develop the social skills of ASD children. Afshari [23] also proposed in the study that consciousness and motor training can effectively strengthen the neurological and cognitive function and improve the attention of ASD children. After evaluating the reaction speed and reaction time of 7 to 10 years old children before and after exercise, Ellemberg and St-Louis-Deschênes [24] presented that physical exercise can improve children's cognitive function. The joint attention behaviour is closely related to social cognition, which can be seen as the first step in the development of children's social cognition. The use of exercise to intervene in children's behaviour should not be limited to existing aspects. There are new and different aspects waiting for being explored. Therefore, exercise as a means of intervention in the joint attention of ASD children is feasible.

Adaptive physical activity is the theoretical support of AGR's design. Adapted physical is a physical activity for people with special needs to achieve the most suitable state between the individual and the environment. Its purpose of is to enable each individual to obtain high-quality sports guidance and participate in sports activities for life [25]. In China, special needs schools are the main compulsory education places for ASD children. They face many cognitive problems. School-based exercise usually includes functional sports or postural tasks, such as squats, sit-ups, long-distance running, and repetitive functional sports. These exercises are not attractive to ASD children and may even be objectionable to young children. Therefore, school-based exercise programmes are necessary. Considering that there are insufficient sports activities suitable for low-functioning ASD children, this research designed adaptive rhythmic gymnastics (ARG). It is based on the basic gymnastics exercises and blended with music, dance, art, play, and other elements. Children can do rhythmic aerobic physical activity under rhythm music. The reasons for choosing gymnastics are as follows:(1) gymnastics require higher concentration to acquire movements, stimulate the development of motor skills, gain health, and promote the quality of life [26]. (2) Gymnastics belongs to aerobic exercise. Strong aerobic capacity also has a positive impact on the cognitive function of developmental disabilities [27, 28]. (3) Rhythm is the natural behaviour of human beings. Rhythmic gymnastics is composed of simple and safe rhythmic movements, which are easy to master and suitable for children with different abilities. Successfully mastering motor skills can help enhance the self-confidence of children with developmental disabilities [29]. (4) Music and game competitions in rhythmic gymnastics can attract children's attention and participation and can also effectively regulate their negative emotions [30]. (5) Gymnastics events do not have high requirements for venues and equipment, which is suitable for special education schools with limited venues. In conclusion, ARG belongs to the category of adaptive sports activities, is based on basic gymnastics, and is a blend of music, dance, games, and other elements; content design is not the purpose of the development of the motor ability of children, but in sports game as the carrier development of children's cognitive ability. For children, ARG is rich in content and attractive. For schools, ARG requires less space than ball games, track and field, and so on. Therefore, AGR is selected as the main intervention method of this study. The purpose of this study is to intervene in two 6-year-old ASD children with AGR to test whether AGR can have a positive effect on the joint attention of young ASD children.

## 2. Method

### 2.1. Participants

Selection criteria: Two ASD children from Peizhi schools were selected as intervention participants. The selection criteria were as follows: (1) ASD children who met the diagnostic criteria for autism spectrum disorder in the fifth edition of the American Diagnostic and Statistical Manual of Mental Disorders Children. (2) Children, with no heart, respiratory, or other diseases, have certain verbal communication skills. The gross motor function part of the Peabody Developmental Movement Scale (PDMS-2) was used for evaluation. PDMS-2 has good reliability and validity in assessing the level of motor development of children aged 0 to 6 years old. Since both participants have intellectual disabilities, and the motor development level of the children with intellectual disabilities is roughly 2 to 4 years behind that of the normal ones [31], this study selected the gross motor indicators that are suitable for 48 to 72 months in PDMS-2 for evaluation. It includes 3 parts: posture control ability, movement ability, and physical operation skills. The test indicated that the gross motor ability and cognitive ability of the two participants were delayed, and there was no big difference between their levels of them. Hence, they were suitable for participating in the AGR course. As the PDMS-2 test was only used to select participants, its data analysis is not included in this study. (3) The parents of the two participants were aware of and agreed with their children taking part in the research. According to observation and interviews, the descriptive characteristics of the participants are illustrated in Table 1.

### 2.2. Measures

#### 2.2.1. Coding Table of Frequency of Joint Attention and Negative Emotion

The researcher created it originally and recorded the results from the course videos.

*(1) Intervention Loyalty Checklist*. In order to ensure the correct implementation of the intervention and the internal reliability of the research, the researcher designed the intervention loyalty checklist initially to evaluate the extent to which the intervention implemented by the teacher is consistent with the original plan (including the objective, content, and implementation of the intervention). The raters were those who had not participated in the experiment and had received loyalty rating training. In this study, 25% of all intervention periods were selected for investigation.

### 2.3. Procedures

This study applied the A-B-A cross-subject multibaseline design in a single case research design. Based on the most basic A-B design, A (baseline period) was replicated. The entire experimental process included three stages, followed by baseline (A1), intervention (B1), and baseline (A2). According to the curriculum arrangement and class activities of the two participants, they were trained individually in the classrooms, which they were familiar with. This was done three times a week for a duration of 50 minutes each time, and the experiment time was carried out for three months in total. (It took 2 weeks for A1, 8 weeks for B2, and 2 weeks for A2.)

### 2.4. Dependent Variables and Measurements

The dependent variable is the joint attention of the research participants. The course videos were coded according to the self-designed “Coding Table of Frequency of Joint Attention” and “Coding Table of Frequency of Negative Emotion.” The coding criteria are as follows:

1. Target behaviour 1 was defined as follows: (1) responding to the impunity, instructions, and gaze behaviour initiated by others and taking the initiative to look at the teacher's eyes (or the upper part of the face); (2) actively present your ideas to others, pointing with their finger to objects or events, displaying objects, or alternately looking at the teacher, the objects, or events. When measuring, the frequency of joint assessment was recorded. (3) Only children's target behaviors related to the intervention content were recorded For example, children do not participate in the intervention activities but go to the teacher to play with their toys.
2. Target behaviour 2 was defined as follows: emotional instability in class, yelling, crying and screaming, crying by hitting the jaw with objects or hands, lack of concentration, and running around.

### 2.5. Independent Variable and Intervention Method

The independent variable was the intervention plan of adaptive rhythmic gymnastics (ARG). Throughout the research process, the researcher designed four sets of rhythmic gymnastics exercises and functional training exercises based on the development level of the participants. During the teaching process, as the participants mastered the movements, the teacher would gradually increase the difficulty of the movements. The content and functions of ARG are demonstrated in Table 2.

### 2.6. Procedure

The entire study was implemented one-on-one by the researcher in classrooms that the participants were familiar with. The intervention procedures of ARG are displayed in Table 3.

Interventions are divided into baseline, implementation, and maintenance phases, each with a different task objective: for example, the baseline objective is to determine the child's baseline level. The total duration is three months:

  Baseline (A1): during this baseline period, the researcher collected the baseline data of children's joint attention through the rhythm games between the teacher and children. The teacher played upbeat music with a strong sense of rhythm and invited participants to do some fundamental rhythmic gymnastics exercises with it. During the practice, the teacher created opportunities for children to initiate joint attention behaviours (such as imitating various animal behaviours and pointing their fingers at the toys that children are interested in). When participants gave an incorrect response, the teacher did not prompt them or intervene. After the joint attention of the test reached a stable level, the baseline data was generated. The measurement was performed 3 times a week. H baseline data was collected for 2 weeks, with a total of 6 data points; baseline data lasted for 3 weeks, with a total of 9 data points.
  Intervention (B1): according to the development level of participants, the researcher created rhythmic gymnastics and exercise games. Under the guidance of the teacher and the teaching assistant, the movements were taught to participants, ranging from simple to difficult, with accompanying music. Participants could respond correctly to the rhythm of the music or the teacher's instructions. During the intervention, the teacher gave hints to participants and provided assistance in initiating or responding correctly when they found it difficult to do so. During the intervention, the number of teachers' help and prompts was gradually reduced according to the state of the child, and the prompts gradually diminished. After the children gave the correct responses (assistant and independent), the teacher immediately strengthened it. In this study, a combination of social reinforcers and operational reinforcers was used. Social reinforcers mainly included verbal praise (such as “XX is awesome!”), thumbs up (the teacher's thumb pointed to the child's thumb), and high-five; operational reinforcers mainly involved the use of stickers that participants were interested in and other toys that children required.
  Baseline (A2): after the intervention task (B1) was completed, remove any intervention with AGR and observe the child's behaviour level for two weeks after discontinuing the intervention. The method of data collection during the second baseline period was the same as that during the first baseline period. When the teacher and children took part in the rhythmic gymnastics exercises, the frequency of joint attention behaviour of children was observed and recorded. Similarly, the teacher neither intervened in the children's joint attention behaviour at this stage nor did they give prompts or reinforcers.

### 2.7. Reliability and Validity

#### 2.7.1. Observer Consistency

Thi**s** study applied the frequency ratio method that was used to guarantee the observer consistency. In each stage of the research process, 30% of the intervention videos were randomly selected. Target behaviours were independently recorded by two trained observers following the same measurement procedure. IOA index is shown in Tables 4–7.

It can be seen from Tables 4–7 that IOA scores were over 85% and met the requirement in this study.

### 2.8. Intervention Loyalty

In order to ensure the correct implementation of the intervention and the internal reliability of the research, this study chose 25% of all intervention periods for the investigation to assess the intervention loyalty. The formula of intervention loyalty percentage is as follows: correct steps/total steps × 100%. In this study, the intervention loyalty was 92%, 93%, 95%, 93%, 96%, 92%, and 94%, which met the requirement of it higher than 90%.

### 2.9. Data Analysis

The frequency of children's joint attention during the entire research process was drawn into a line chart. In addition, the visual analysis method is applied to analyse and compare the changes in children's joint attention within and between stages of the study. At the same time, the Tau effect value calculation method proposed by Parker, Vannest, Davis, and Sauber [32] was used to calculate the intervention effect of two participants. As a nonparametric test method, a pair-to-pair comparison was made of each data point in baseline period A with each data point of interventional expectation B to obtain the number of overlapping data pairs (B ˂ A), nonoverlapping data (B>), and equal data pairs (*A* = *B*). The intervention effect value was calculated by the formula: Tau = (the number of nonoverlapping data pairs − the number of overlapping data pairs)/total amount of data pairs (total amount of data pairs = *nA* × *nB*). If the Tau effect value is under 0.5, it indicates that the intervention effect is insignificant; if the value is between 0.5 and 0.69, it implies that the intervention effect is medium; if the value is within the range of 0.7 to 1, it represents that the intervention effect is significant.

## 3. Results

**Figure 1:**
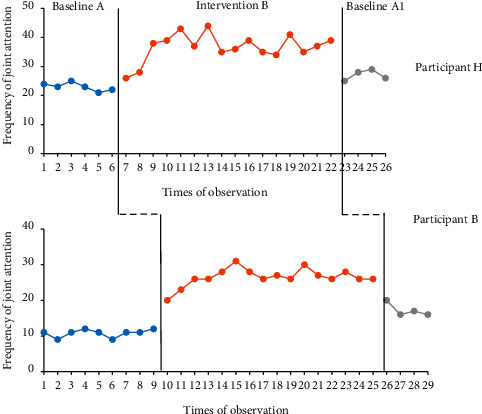
Line chart of joint attention frequency of two ASD children.

This research used ARG to intervene in the joint attention behaviour of two 6-year-old ASD children. The joint attention behaviour of participants was evaluated 26 times (participant H) and 29 times (participant M) in three months. The results are presented in Figure 1.

According to Figure 1, the changes in the joint attention frequency of both ASD children within each stage and cross stages are explained from the perspectives of trends, levels, range of change, and overlap rate.

### 3.1. Analysis of Intrastage Changes in the Joint Attention of Both ASD Children

#### 3.1.1. Baseline A

It can be seen from Figure 1 and Table 8 that during the baseline period A, the joint attention frequency of children H and M ranged between 21 to 25 times and 9 to 12 times, with average levels at 23 and 10.78 times, respectively. The average level of M was lower than that of H's. During this stage, the trend of H slowly declined; however, the trend of M was the opposite. The horizontal stability was 66.7% and 77.8%, respectively. This illustrated that the children's joint attention was relatively stable during this stage.

#### 3.1.2. Intervention B

According to Figure 1 and Table 8, in intervention period 1 (B1), the joint attention intervention of both children has achieved a certain effect. The average level was increased to 36.75 and 26.5 times, which was 13.75 and 16.5 times higher than the baseline period, respectively. The level range was between 20 to 31 and 26 to 44 times, respectively. The horizontal stability at this stage was 62.5% and 75%, respectively, which indicated a moderately strong and unstable upward trend.

#### 3.1.3. Baseline A1

Figure 1 and Table 8 demonstrate that after the intervention of adaptive rhythmic gymnastics (ARG) was removed, the joint attention of these two children returned to near baseline level. Meanwhile, the average level was slightly higher than that of the baseline period A. During the baseline period A1, the joint attention levels of both children ranged between 16 to 20 and 26 to 19 times, and the average level was 27 and 17.25 times, respectively. The horizontal stability was 50% and 66.7%, respectively, which manifested a relatively stable state.

### 3.2. Interstage Change Analysis of Two ASD Children's Joint Attention

#### 3.2.1. Baseline B/Intervention A1

It can be noticed from Figure 1 and Table 9 that when the ARG intervention was implemented, the joint attention of child H increased from 21 to 31 times, and the joint attention of child M increased from 9 to 44. The results disclosed that the intervention had an effect on raising the level of joint attention for both participants, especially for child M. The overlap rate of the data between these stages was 0%, and the Tau effect value was 1, indicating that the intervention had a significant effect on both participants.

#### 3.2.2. Intervention 1/Baseline 2

It can be seen from Figure 1 and Table 9 that after removing the ARG intervention, the level of child H's joint attention dropped rapidly from 31 times to 20 times, and the level of child M's joint attention also declined from 44 times to 26 times. The overlap percentages of secondary intervention period B and baseline period A1 were both 25%, while the Tau effect values were 0.84 and 0.98, respectively. This implied that there was a functional relationship between ARG intervention and children's joint attention.

### 3.3. Analysis of Negative Emotional Behaviour Changes in Two ASD Children

**Figure 2:**
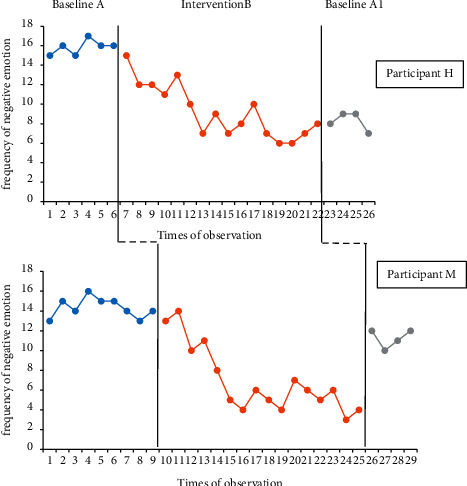
The observation chart of the frequency of two participants' negative emotions.

The number of occurrences of negative emotional behaviour of two participants in baseline period A, intervention expectation B, and baseline period A1 was plotted as a line chart. It is convenient and easy to analyse the changes in the negative emotions of two participants at different stages. The specific analysis is shown in Figure 2.

It can be noticed from the trend in Figure 2 that the number of negative emotions of the two participants showed a downward trend after the intervention. After the intervention was removed, the negative emotions of participant H essentially remained at the same level as in the intervention periods. The increase in the number of negative emotions in participant M was close to the level of baseline A. Although the intervention effects on the two participant's negative emotions were different, the observation data can prove that there is a functional relationship between ARG and the emotional behaviours of the two ASD children.

### 3.4. Social Validity

#### 3.4.1. Comprehensive Analysis of Social Validity Survey Results

The acceptance (*M* = 4.63, SD = 0.55), feasibility (*M* = 4.5, SD = 0.48), and satisfaction (*M* = 4.12, SD = 0.8) scores of the participants' head teachers and their parents were significantly high. This signified that the ARG intervention programme has good social validity.

## 4. Discussion

### 4.1. Adaptive Rhythmic Gymnastics (ARG) Can Effectively Develop the Joint Attention Behaviour of ASD Children

This study adopted a cross-subject multibaseline A-B-A design in a single case research design. Two 6-year-old ASD children were examined through an ARG course to promote the development of their joint attention. Both the analysis of the data and the feedback from the teachers showed the positive effects brought by the course. During the research, when the ARG intervention was introduced, the frequency of children's joint attention grew significantly. However, when the intervention was removed, the joint attention frequency of the two children fell to the baseline level. Consequently, the experiment achieved strict control and revealed that ARG intervention can effectively improve the joint attention of ASD children.

First of all, in the ARG program, language and finger instructions [33] can stimulate the development of children's joint attention. Although this study did not record separately between initiating joint attention and responding to joint attention, it was found during the video observation process that ARG has a better effect on responding joint attention of the two participants than initiating joint attention, which is especially displayed during the early stage of the experiment. This may result from the children's unfamiliarity with the exercise content. Previous studies have suggested that responding joint attention is the basis of initiating joint attention [10]. Besides, the relation between them is ladder-like and coherent. Both RJA and IJA belong to the joint attention category; the latter is more inclined to the active behaviours of the subjects. RJA refers to responding to another person's request for joint attention, and IJA signifies the initiative of joint attention. In contrast, IJA is a child-initiated behaviour, which requires more from children. During ARG intervention, both verbal and nonverbal actions are included, and this is what makes ARG unique: it can express its emotions (positive and negative emotional states) through body movements. Treating the improvement of both as the research goal of joint attention can promote the development of joint attention in ASD children in a more three-dimensional and comprehensive way.

Moreover, the ARG intervention program was designed with elements such as music and games, and a game-based practice method was also used. In previous studies, an ample amount of recent studies applied music therapy as an effective means of developing the joint attention of ASD children [34, 35]. Scholars explored whether the rhythm and structure of music can provide external clues for the organisation, prediction, and response of autistic children and found that it has a unique attraction for these children [36]. Furthermore, imitation is a critical prerequisite for the cognitive development of children [37]; the symbolic ability is a vital component of developing the joint attention of children [38]; and animal imitation exercises in ARG can exercise the symbolic ability of children. Game-based ARG intervention can bring entertainment to exercise. Without specific technical requirements, simple and variable games are very effective intervention methods for young children. It can attract children's attention and stimulate interaction between teachers and students. More importantly, it can develop children's cognition and creativity [39, 40].

Finally, during the intervention process, the teacher gradually increased the difficulty of the action according to the children's learning situation. Strictly controlling the intensity during the exercise [41] proposed that individuals with developmental disabilities should exercise at 65% to 70% of the maximum heart rate to achieve good results and can avoid the phenomenon that exercise intensity is too high to produce exercise fatigue or exercise intensity is too low to reduce children's interest in learning. Ultimately, achieve a good intervention effect. Therefore, it is very necessary to set reasonable exercise intensity, which can affect the final intervention effect on children and is an important link that cannot be ignored in the design of an intervention program. The exercise intensity of ARG should be scientifically arranged according to the characteristics of the subjects. The common attention ability behind the exercise should not be neglected because the intensity is too high, and the ability development of children should not be stopped because the intensity is too low.

### 4.2. Adaptive Rhythmic Gymnastics (ARG) Can Improve Emotional Problems of ASD Children in a Certain Extent

Based on the statistics on children's emotional problems, it can be disclosed that ARG can improve children's negative emotions. After the intervention, the interview with the parents of participants demonstrated that ARG can seriously improve participants' negative emotional behaviours. More importantly, it provided parents with a new way to communicate with their children and alleviate their children's emotional problems.

Studies have found that play and music have great appeal to children. Music, especially, can alleviate children's negative emotions to a certain extent [42]. Although they have emotion control disorders in their daily lives, they can properly perceive emotions through music [43]. When the music started to play, the children focused on the teacher and started to move their bodies with the music spontaneously. Subsequently, their emotions would gradually stabilise.

Meanwhile, effective “communication” is central to the improvement of children's negative emotions. Children with autism tend to have language and social communication barriers. Their behaviour and communication are directly related [44]. It has been stated in many studies that when providing education and treatment for ASD children, communication between caregivers and children is the main focus [45–47]. In addition to in the home, schools are the most important activity places for children. Teachers play a critical role [48, 49], and therefore, interaction with children can have a profound impact on them. The results of this research support this point of view. Before implementing the ARG intervention program, the general basic information of the two children have been thoroughly examined. During the implementation process, the program was adjusted immediately by observing children's reactions. Teachers showed their enthusiasm and friendliness to the children and encouraged them while they practised movements and played games. In the fun and relaxed learning atmosphere, children's negative emotions were stabilised and their emotional control ability saw improvement.

### 4.3. Limitations

First of all, this research is a single case research with a small number of participants. The generalisation of results is relatively weak. Follow-up research may adopt the educational experiment paradigm and set up the experimental group and the control group for large sample intervention. A follow-up study can be addressed on current participants, while more eligible children can be recruited to get involved in this program. Accumulating samples semester by semester, the sample size will increase not only in number but also in the efficiency of the intervention. Moreover, since the duration of the intervention was short, the participants did not form a good generalisation and the effect of the follow-up intervention faded quickly. Hence, the results of the intervention have certain limitations. Furthermore, although the frequency of children's joint attention was significantly increased, the positive effects of the quality of joint attention have not been discussed in depth. In subsequent studies, the increase in both quantity and quality needs to be comprehensively considered.

## Figures and Tables

**Table 1:** Descriptive characteristics of participants.

Name	M	H
Gender	Male	Male

Age	6	6

Cognitive level	1. Know the numbers up to 10 and understand the basic colours and shapes.	1. Know the numbers up to 10 and understand the basic colours and shapes
	2. A gerund instruction and a simple two-step instruction could be executed. However, complex two-step instructions and instructions with more steps could not be executed accurately.	2. A gerund instruction and a simple two-step instruction could be executed. Complex two-step instructions and multistep instructions could be executed accurately once in a while with the teacher's multiple repetitions
	3. Attention was easily distracted, and the duration was short. It was difficult to participate in class activities during group class. When in a good mood, he was capable of participating in class activities under the leadership of the teacher	3. Attention was easily distracted, and the duration is short. Nevertheless, he was capable of participating in classroom activities under the leadership of the teacher

Social interaction level	He could look into others' eyes for a short time and simply respond to them when his name was called. However, he could not actively initiate social behaviour and take the initiative to greet the teacher. He has weak joint attention.	He gave a response and gaze when his name was called. He took the initiative to socialise with others occasionally and recognised the teacher. However, he did not initially greet others. He has weak joint attention.

Emotional problem behaviour	Emotionally unstable, yelling, hitting the jaw with objects or hands	Emotionally unstable, inattention, crying and screaming, running around

Reinforcer	Beautiful stickers, picture books	Electronic devices

**Table 2:** The ARG program contents and functions.

Program categories	Contents	Functions
Rhythmic gymnastics exercises	1. Hand clapping exercises (including rhythmic stepping, clapping hands, foot-touching, and shoulder clapping during travel) 2. Little frog exercises (including imitating frogs, bending and extending knees, squatting up, switching balance jumps on one foot, and so on) 3. Little bunny exercises (including both hands imitating rabbit ears, continuous vertical and horizontal jumping in different directions) 4. Goose balance exercises (including one-legged balance standing, weight shifting on both feet, both hands side-level lift up and down with rhymes)	1. Develop large muscle groups from motor skills, promote nerve-muscle coordination, and enhance body control and displacement capabilities. Evoke automatic posture control through vertical and horizontal jumping. Combine auditory, tactile, visual, and vestibular stimulation to achieve visual and kinesthetic stimulation and strengthen balance control ability. 2. Clapping exercises can facilitate the interaction and communication between teachers and students, and increase their interest in the classroom. 3. Learn animal imitation exercises, understand the teacher's instructions, and attract children's attention. Improve children's imitation ability and develop children's cognitive ability.

Functional training exercises	Strength, endurance, flexibility, agility, and coordination exercise	Improve the physical fitness and develop the physical health of children.

**Table 3:** The implementation procedure of the ARG programme.

Stages		Activities	Time (minutes)
Beginning section	Routine	Teachers greeted participants and told them the aim of this lesson was to promote the relationship between teacher and student.	8
	Warm-up	Children followed the rhythm of medium-speed music for warm-up (static and dynamic stretching, marking time, and joint mobility) to increase muscle temperature, prevent injury, and gradually enter a learning state.	

Fundamental section		1. Under the guidance of teachers, children followed the rhythm of the music and learned the movement sets of rhythmic gymnastics (clapping hands, frogs, rabbits, and wild geese exercise). 2. Children imitated all kinds of small animals (frogs, rabbits, elephants, and so on.) to learn the movements of climbing, jumping, and rolling, among others. Additionally, they completed mirror imitation, speed competitions, and other games with teachers. 3. Children performed 10 times*∗*2 groups of sit-ups and 8 times*∗*2 groups of supine leg lifts to develop children's waist and abdomen strength.	35

Ending section	Cool-down	Children performed dynamic and static stretching and adjusted their breathing to calm the body.	5
	Summary	The teacher summarised the children's performance and praised their good behaviour before saying goodbye to each other.	2

**Table 4:** IOA index of joint attention of participant M.

Frequency	1	2	3	4	5	6	7	8	9
Observer 1	12	11	22	27	30	27	30	22	21
Observer 2	11	11	20	28	31	26	32	20	20
IOA (%)	91.6	100	90.9	96.4	96.7	96.3	93.7	90.0	95.2

**Table 5:** IOA of joint attention of participant H.

Frequency	1	2	3	4	5	6	7	8	9
Observer 1	24	22	23	28	38	40	36	35	24
Observer 2	22	23	22	26	39	41	34	36	26
IOA (%)	91.7	95.7	95.7	92.9	97.4	97.6	94.4	97.2	92.3

**Table 6:** IOA of negative emotion of participant M.

Frequency	1	2	3	4	5	6	7	8	9
Observer 1	13	15	10	11	5	7	6	5	7
Observer 2	14	14	11	9	5	6	7	5	8
IOA (%)	92.6	93.3	91	81.8	100	85.7	85.7	100	85.7

**Table 7:** IOA of negative emotion of participant H.

Frequency	1	2	3	4	5	6	7	8	9
Observer 1	16	15	17	15	10	8	6	9	14
Observer 2	15	16	16	15	11	7	7	8	13
IOA (%)	93.8	93.8	94.1	100	91	85.7	85.7	88.9	92.6

**Table 8:** Analysis of intrastage changes in the joint attention of both ASD children.

Experimental stages	Baseline a		Intervention B		Baseline A1	
	Participant H	Participant M	Participant H	Participant M	Participant H	Participant M
Trend	\	/	/	/	/	\
Range	21–25	9–12	20–31	26–44	16–20	26–29
Change level	2(−)	1(+)	3(+)	6(+)	1(−)	4(−)
Mean	23	10.78	36.75	26.5	27	17.25
Horizontal stability	66.7%	77.8%	62.5%	75%	50%	75%

**Table 9:** Interstage change analysis of the joint attention of both ASD children.

Experimental stages	Baseline (A)/intervention (B)		Intervention (B)/baseline (A1)	
	Participant H	Participant M	Participant H	Participant M
Changes in effect	Positive	Positive	Positive	Positive
Ranges	21–31	9–44	20–31	26–44
Overlap percentage	0%	0%	25%	25%
Tua value	1	1	0.84	0.98

## Data Availability

The data sets generated and/or analysed during the current study are available from the corresponding author on reasonable request.
